# 5-Hydroxymethylcytosine signatures in circulating cell-free DNA as diagnostic biomarkers for human cancers

**DOI:** 10.1038/cr.2017.121

**Published:** 2017-09-19

**Authors:** Wenshuai Li, Xu Zhang, Xingyu Lu, Lei You, Yanqun Song, Zhongguang Luo, Jun Zhang, Ji Nie, Wanwei Zheng, Diannan Xu, Yaping Wang, Yuanqiang Dong, Shulin Yu, Jun Hong, Jianping Shi, Hankun Hao, Fen Luo, Luchun Hua, Peng Wang, Xiaoping Qian, Fang Yuan, Lianhuan Wei, Ming Cui, Taiping Zhang, Quan Liao, Menghua Dai, Ziwen Liu, Ge Chen, Katherine Meckel, Sarbani Adhikari, Guifang Jia, Marc B Bissonnette, Xinxiang Zhang, Yupei Zhao, Wei Zhang, Chuan He, Jie Liu

**Affiliations:** 1 Department of Digestive Diseases, Huashan Hospital, Fudan University, Shanghai 200040, China;; 2 Section of Hematology/Oncology, Department of Medicine, University of Illinois, Chicago, IL 60612, USA;; 3 Department of Chemistry, Department of Biochemistry and Molecular Biology, and Institute for Biophysical Dynamics, Howard Hughes Medical Institute, The University of Chicago, Chicago, IL 60637, USA;; 4 Shanghai Epican Genetech, Co. Ltd., Zhangjiang Hi-Tech Park, Shanghai 201203, China;; 5 Department of General Surgery, Peking Union Medical College Hospital, Chinese Academy of Medical Sciences, Beijing 100730, China;; 6 Department of General Surgery, Huashan Hospital, Fudan University, Shanghai 200040, China;; 7 Department of Integrative Oncology, Shanghai Cancer Center, Fudan University, Shanghai 200032, China;; 8 Department of Digestive Diseases, Pudong Hospital, Fudan University, Shanghai 201399, China;; 9 Department of Oncology, Nanjing Drum Tower Hospital, Nanjing University Medical School, Nanjing 210008, China;; 10 Beijing National Laboratory for Molecular Sciences, College of Chemistry, Peking University, Beijing 100871, China;; 11 Key Laboratory of Bioorganic Chemistry and Molecular Engineering of Ministry of Education, Peking University, Beijing 100871, China;; 12 Department of Medicine, The University of Chicago, Chicago, IL 60637, USA;; 13 Department of Chemical Biology, Structure and Function Biomolecules Center, College of Chemistry and Molecular Engineering, Peking University, Beijing 100871, China;; 14 Department of Preventive Medicine and The Robert H. Lurie Comprehensive Cancer Center, Northwestern University Feinberg School of Medicine, Chicago, IL 60611, USA;; 15 Department of Immunology, State Key Laboratory of Genetic Engineering, Institutes of Biomedical Sciences, Fudan University, Shanghai 200433, China

**Keywords:** 5-hydroxymethylcytosine, liquid biopsy, circulating cell-free DNA, cancer biomarker, 5hmC-Seal

## Abstract

DNA modifications such as 5-methylcytosine (5mC) and 5-hydroxymethylcytosine (5hmC) are epigenetic marks known to affect global gene expression in mammals. Given their prevalence in the human genome, close correlation with gene expression and high chemical stability, these DNA epigenetic marks could serve as ideal biomarkers for cancer diagnosis. Taking advantage of a highly sensitive and selective chemical labeling technology, we report here the genome-wide profiling of 5hmC in circulating cell-free DNA (cfDNA) and in genomic DNA (gDNA) of paired tumor and adjacent tissues collected from a cohort of 260 patients recently diagnosed with colorectal, gastric, pancreatic, liver or thyroid cancer and normal tissues from 90 healthy individuals. 5hmC was mainly distributed in transcriptionally active regions coincident with open chromatin and permissive histone modifications. Robust cancer-associated 5hmC signatures were identified in cfDNA that were characteristic for specific cancer types. 5hmC-based biomarkers of circulating cfDNA were highly predictive of colorectal and gastric cancers and were superior to conventional biomarkers and comparable to 5hmC biomarkers from tissue biopsies. Thus, this new strategy could lead to the development of effective, minimally invasive methods for diagnosis and prognosis of cancer from the analyses of blood samples.

## Introduction

Cytosine methylation (formation of 5-methylcytosine, 5mC) is a well-established epigenetic mechanism that affects global gene expression^1,2^. The 5mC remodeling of DNA is used extensively during mammalian development and cell differentiation, as well as during cancer initiation, progression and in the therapeutic response^3,4^. Active demethylation in the mammalian genome is mediated by the TET (Ten-Eleven Translocation) family of dioxygenases that oxidize the 5mC modification to 5-hydroxymethylcytosine (5hmC)^5,6^, and further to 5-formylcytosine (5fC), and 5-carboxylcytosine (5caC)^7,8,9^. The “intermediate” 5hmC not only marks active demethylation but also serves as a relatively stable DNA mark that plays distinct epigenetic roles^2,10,11,12,13,14,15^. Recent genome-wide sequencing maps of 5hmC in various mammalian cells and tissues support its role as a marker for gene expression^16,17,18,19,20,21,22^; it is enriched in enhancers, gene-bodies and promoters, and changes in 5hmC correlate with changes in gene expression levels^22,23^.

The discovery of cell-free DNA (cfDNA) originating from different tissues in the circulating blood has revolutionary potential for the clinic^24^. Liquid biopsy-based biomarkers and detection tools offer substantial advantages over existing diagnostic and prognostic methods, including being minimally invasive. They thus have a cost-efficient potential to promote higher patient compliance and clinical convenience to enable dynamic monitoring^25^. Tumor-related somatic mutations in cfDNA have been shown to be shared with the tumor tissue, although low mutation frequency and the lack of information on tissue of origin hamper the detection sensitivity. 5mC and 5hmC in cfDNA from liquid biopsies could serve as parallel or more valuable biomarkers for non-invasive diagnosis and prognosis of human diseases, because they recapitulate gene expression changes in relevant cell states. If these cytosine modification patterns can be sensitively detected, disease-specific biomarkers could be identified for effective early detection, diagnosis and prognosis.

High-throughput sequencing is an ideal platform for detecting genome-wide cytosine modification patterns. Whole-genome bisulfite sequencing or alternative reduced representative methods have been applied in biomarker research with cfDNA^26,27,28^. Tissue- and cancer- specific methylation sites have shown promising performance in tracking tissue-of-origin from circulating blood^26,28^. However, 5mC serves mostly as a repressive mark with a high background level in the human genome, and its sequencing with bisulfite treatment has been hampered with extensive DNA degradation, in particular with cfDNA. Taking advantage of the presence of the hydroxymethyl group, selective chemical labeling can be applied to map 5hmC using low levels of DNA with high sensitivity. The profiling method is robust and cost-effective for studies of large cohorts and practical applications. Here, we have established 5hmC-Seal technology for 5hmC profiling in cfDNA. We show that the differentially enriched 5hmC regions in cfDNA are excellent markers for solid tumors.

## Results

### 5hmC-Seal profiling in clinical specimens

**Figure 1:**
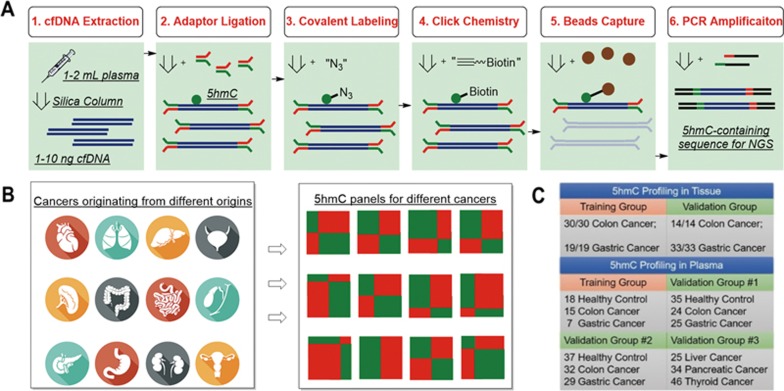
Detecting 5hmC biomarkers in cfDNA of human cancers. **(A)** Workflow of 5hmC-Seal profiling from cfDNA is shown. Purified cfDNA is ligated with standard sequencing adaptors. 5hmC-containing cfDNA fragments are selectively labeled with a biotin group. The biotin-labeled fragments are captured on the avidin beads, followed by PCR amplification and next-generation sequencing (NGS). **(B)** Cancers of different tissue origins (e.g., lung, colon, stomach, liver, ovary, pancreas) may release cfDNA decorated with distinct 5hmC modification patterns. Unique 5hmC signatures specific for different cancer types could be detected as biomarkers for diagnosis and prognosis. As shown in this panel, a unique 5hmC signature may correspond to each cancer type. **(C)** Schematic overview of sample collection, data generation and analysis is shown.

We first optimized our previously published nano-hmC-Seal profiling method^29^ (Figure 1) for cfDNA. The adaptor was pre-ligated with barcodes to enhance the efficiency of library construction and decrease cross contamination between large cohorts of samples. The labeling, binding and washing steps were optimized for capturing limited 5hmC-containing cfDNA fragments. We profiled 5hmC in plasma cfDNA from cancer patients and healthy controls, as well as in genomic DNA (gDNA) isolated from tumors and adjacent healthy tissues. We sampled 90 healthy individuals, 260 cancer patients and 71 patients with benign diseases among Chinese populations (Supplementary information, Tables S1 and S2). For these patients and healthy controls, the study generated 401 5hmC-Seal libraries from plasma cfDNA and 192 5hmC-Seal libraries from tissue gDNA (Supplementary information, Table S3). The cohort samples were collected and profiled in three batches (Supplementary information, Table S3). To minimize the influence of an experimental batch effects, differential 5hmC between cancers and controls (or between tumors and adjacent tissues) was analyzed with the first (discovery) batch and validated in the second (validation) and third (additional validation) batches.

To validate the 5hmC capture efficiency and the reliability of the modified assay, we spiked a pair of synthetic DNA probes containing and not-containing 5hmC into plasma cfDNA. The 5hmC-Seal capture generated an average of 56-fold 5hmC enrichment of the spiked-in probes compared to control without pull-down (Supplementary information, Figure S1A). Samples with physiologically relevant amounts of cfDNA (1, 2, 5, 10, 20 ng) and 5hmC-containing spike-in probes at a fixed amount of 2.6 fg were processed and sequenced, respectively. A linear relationship was observed between the proportion of 5hmC-containing spike-in readouts and the spike-in concentration within cfDNA (*r*^2^ = 0.99, Supplementary information, Figure S1B), confirming the quantitative capture of 5hmC even down to 1 ng of input cfDNA.

### Global and genomic distribution of 5hmC modifications

We evaluated the global 5hmC level variation in cancer by using ultra-sensitive capillary electrophoresis-electrospray ionization-mass spectrometry (CE-ESI-MS)^30^. Global 5hmC levels of the tumor gDNA were markedly decreased compared to the adjacent healthy tissue gDNA, with an average of 85% and 64% reduction in colorectal and gastric tumor samples, respectively. Global 5hmC levels of the cancer patients' plasma cfDNA showed a more limited decrease compared to control plasma cfDNA, consistent with low proportions of tumor-derived DNA in the total cfDNA pool (Supplementary information, Figure S2).

In plasma cfDNA, 5hmC was enriched within gene bodies and DNase I sensitive peaks whereas it was depleted at transcription start sites, CpG islands and transcription factor (TF) binding peaks relative to the flanking areas (Supplementary information, Figure S3A-S3F). This suggests an accumulation of 5hmC surrounding TFs at active transcription sites. 5hmC was also enriched in regions bearing permissive histone marks such as H3K27ac, H3K4me1 and H3K9me1, whereas it was underrepresented in regions bearing repressive markers such as H3K9me3 (Supplementary information, Figure S3G-S3R). The genomic enrichment pattern of 5hmC was consistently observed in tissues gDNA (Supplementary information, Figure S3) and was similar between disease and healthy samples (Supplementary information, Figure S4).

### Differential 5hmC loci associated with colorectal cancer

**Figure 2:**
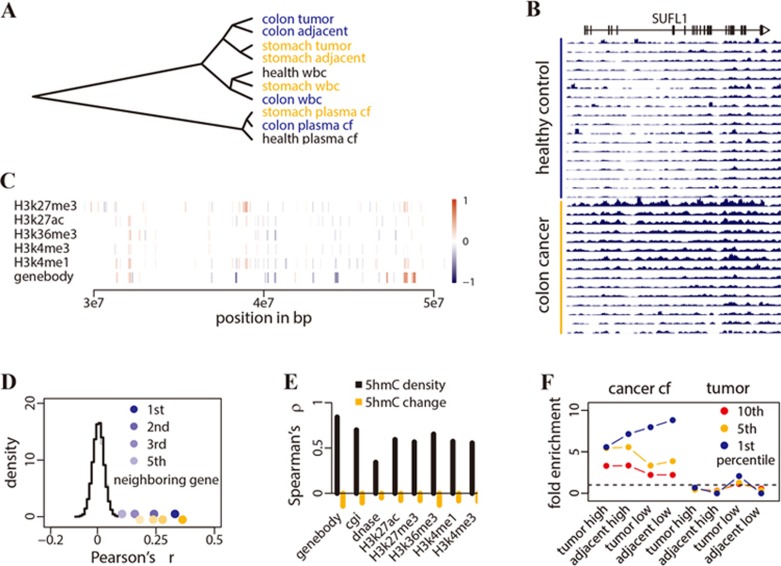
Differential 5hmC loci associated with cancer. **(A)** Average 5hmC levels in gene bodies in healthy controls (health) and cancer patients (colon, stomach), estimated for plasma cfDNA (plasma cf), white blood cell genomic DNA (WBC) and tissue genomic DNA (tumor, adjacent), were clustered by correlation distance. **(B)** Counts per million reads at *SULF1* gene (plus ± 20 kb region) in plasma cfDNA of the 15 healthy controls and 18 colorectal cancer patients. The moving averages at 0.01 smoother span are shown. **(C)** The distribution of colorectal cancer-associated 5hmC loci detected at 5% false discovery rate in plasma cfDNA. Each vertical bar denotes a differential locus (a histone modification peak or a gene body). The color key indicates the relative magnitude of log_2_ fold change in cancer patients vs controls. **(D)** Pearson's correlation of log_2_ fold changes between all analyzed genes and their neighboring genes (points) was plotted against the null distribution of correlation with their first neighboring genes (curves), generated by shuffling gene positions for 1 000 times. Blue and orange points denote data from plasma cfDNA and tissue gDNA, respectively, for colorectal cancer. In **C** and **D**, chromosome 1 is shown as an example. **(E)** Cancer plasma cfDNA and tumor gDNA exhibit correlation in average 5hmC density (library size and feature length normalized log_2_ counts, black bars). However, there is no correlation in the log_2_ fold changes between differential 5hmC loci detected (between cancer vs health (plasma cfDNA)) and tumor vs adjacent tissue (tissue gDNA), (orange bars). **(F)** Genes with a 5hmC level elevated in cancer plasma cfDNA (cancer cf) are enriched in genes with high 5hmC level in tissue gDNA (tumor high, adjacent high). To estimate fold enrichment, the 1st, 5th and 10th percentile genes in descending order of the log_2_ fold change in cancer cfDNA were compared against the corresponding percentile genes in descending order of the average 5hmC level in tissue gDNA. Similarly, genes with a 5hmC level decreased in cancer plasma, and cfDNA are enriched in genes with low 5hmC levels in tissue gDNA (tumor low, adjacent low). In contrast, differentially marked genes detected in tumor gDNA (tumor) show no such enrichment pattern. Dashed line denotes no enrichment.

The average 5hmC profiles of plasma cfDNA were distinct from those of tissue and blood cell gDNA (Figure 2A), which could be due to their distinct cell origins and/or the different DNA degradation properties in cell-free circulation. Among gDNA profiles, variations attributable to tissue identity (colon and stomach tissues, white blood cells) were dominant over variations attributable to disease status (healthy individual vs cancer patient, tumor vs adjacent tissue). Across plasma cfDNA profiles, colorectal and gastric cancer samples were more closely related with each other than with healthy controls (Figure 2A).

We compared 5hmC profiles from plasma cfDNA between 15 colon cancer patients and 18 healthy controls in the discovery batch to identify differentially modified 5hmC loci. The profiles were separated into 18 feature categories: gene bodies, promoters, CpG islands and *cis*-regulatory elements delineated by the Encyclopedia of DNA Elements (ENCODE)^31^. A parallel analysis compared 5hmC profiles from gDNA between colorectal tumors and adjacent tissues in 30 patients in the tissue discovery batch. All feature categories showed enrichment of differentially modified 5hmC loci (Supplementary information, Table S4). Figure 2B shows a differential locus detected in plasma cfDNA at the *SULF1* (sulfatase 1) gene. In cancer patients' plasma cfDNA, the 5hmC levels in *SULF1* were elevated in both exons and introns, with a peak pattern similar to that of tissue gDNA (Supplementary information, Figure S5). Neighboring differential 5hmC loci showed regionally elevated or decreased 5hmC levels (Figure 2C). For example, the correlation of cancer-associated 5hmC changes between neighboring genes was significantly higher than a null distribution generated by shuffling gene positions within a chromosome (Figure 2D). This may suggest that 5hmC modifications occur and change in a relatively long-range, region-wise pattern.

The 5hmC densities across genomic features showed a correlation between cancer plasma cfDNA and tumor gDNA samples (Spearman's *ρ* 0.34-0.84, Figure 2E). This locus-specific correlation of the 5hmC density is expected because of biological constraints. In contrast, we found no correlation between cancer patients' plasma cfDNA and tumor gDNA for cancer-associated 5hmC changes (Figure 2E). This can be explained by the greater variation in 5hmC levels among different tissues than that between disease status (Figure 2A). When gDNA from tumor tissue is released into plasma and mixed with the vast amount of background cfDNA derived from a variety of different tissues, the additional tumor signal observed at a given locus would be determined by the order of locus-, tissue- and disease-specific variations. Consistent with this expectation, we found that genes with 5hmC level elevated in cancer patients' plasma cfDNA were enriched in genes with high 5hmC levels in the tumor tissue gDNA. The top 1% of genes with the most elevated 5hmC levels in cancer patients' plasma cfDNA were enriched by over five-fold in the top 1% of genes with the greatest 5hmC levels in tumor and adjacent tissues (Fisher's exact tests *P* < 0.001). Similarly, genes with 5hmC levels decreased in cancer patients' plasma cfDNA were enriched in genes with low 5hmC levels in tumor and adjacent tissues (Figure 2F). In contrast, no such enrichment pattern was observed for the differentially modified 5hmC loci detected in tumor gDNA (Figure 2F).

### Classification of colorectal cancer by 5hmC markers derived from plasma cfDNA

**Figure 3:**
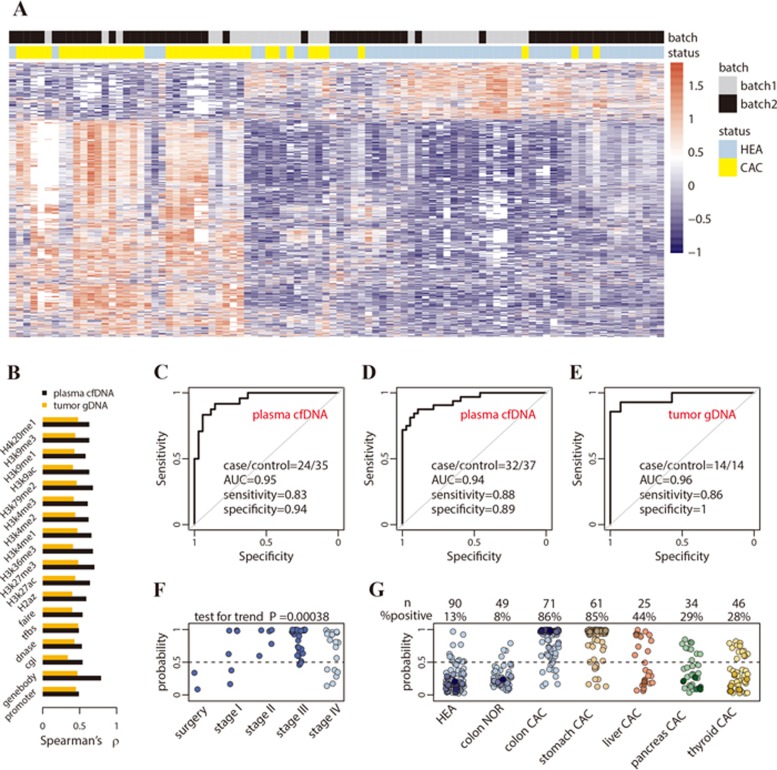
Performance of 5hmC biomarkers for colorectal cancer. **(A)** The heat map shows clustering of cfDNA samples from both the discovery and validation batches, using the 989 differential gene bodies detected in plasma cfDNA from the discovery batch. **(B)** Correlation of 5hmC changes in cancer between the discovery and validation batches is higher in plasma cfDNA (cancer patients vs healthy individuals) than in tissue gDNA (tumors vs adjacent tissues), especially for 5hmC in gene bodies. **(C**, **D)** Classification of two independent validation batches using 5hmC classifier derived from plasma cfDNA from the discovery batch. **(E)** Classification of an independent set of colon cancer tumor tissues using 5hmC biomarkers detected from the discovery batch of tissue samples (tumors vs adjacent tissues). **(F)** The predicted cancer probability (i.e., score) based on 5hmC classifier from plasma cfDNA shows a significant trend associated with clinical stage. Patients after surgery show predicted scores undistinguishable from healthy individuals. **(G)** The 5hmC cfDNA classifier for colorectal cancer is disease- and potentially cancer type-specific, showing decreasing predicted probability in cfDNA from stomach, liver, pancreatic and thyroid cancer patients. AUC, area under curve; CAC, cancer patients; HEA, healthy controls; NOR, patients with benign tumor.

Unsupervised hierarchical clustering using differentially modified 5hmC loci derived from plasma cfDNA generally separated colorectal cancer patients from healthy individuals in the validation batch (Figure 3A). Across the various feature categories, the log_2_ fold change of 5hmC levels in gene bodies showed the greatest correlation between the discovery and validation batches (Spearman's *ρ* = 0.79, Figure 3B), indicating that 5hmC loci in gene bodies are potentially more stable cancer biomarkers. We selected 989 differential loci in gene bodies detected at a 5% false discovery rate (FDR) with a greater than 1.2-fold change (increase or decrease in cancer; Supplementary information, Table S5) for cancer classification. A model-based classifier that applies elastic net regularization on logistic regression was trained using the discovery samples (15 patients vs 18 controls) and then tested in the validation samples (24 patients vs 35 controls). Receiver operating characteristic (ROC) curves were generated to evaluate the performance using the area under the curve (AUC). The prediction algorithm achieved 83% sensitivity and 94% specificity (AUC = 0.95, Figure 3C) for patient classification.

An additional validation batch (32 patients vs 37 controls) was independently collected and tested, achieving 88% sensitivity and 89% specificity (AUC = 0.94, Figure 3D). The classifier was further tested on a set of US samples (Supplementary information, Table S2), all of European descent, collected at the University of Chicago Medical Center. The classifier detected 4 out of 5 patients as cancer positive (80% sensitivity) and called 1 out of 6 healthy controls (83% specificity) in this small cohort. Therefore, although the classifier was trained on Chinese patients, it could capture a general signal of 5hmC changes in plasma cfDNA in colorectal cancer.

For comparison, we applied similar approaches to evaluate the performance of 5hmC biomarkers derived from colorectal tumor tissues. We selected 219 differential loci at gene bodies called at 5% FDR and 1.2-fold change between tumor and adjacent tissues (Supplementary information, Table S6) from 30 patients of the discovery batch. The 5hmC tissue biomarkers showed a sensitivity of 86% and a specificity of 100% (AUC = 0.96, Figure 3E) in 14 patients from the tissue validation batch, suggesting that the 5hmC biomarkers from plasma cfDNA exhibit performance comparable to that from tissue gDNA. The colorectal cancer classifiers derived from plasma cfDNA and tissue gDNA profiles are detailed in Supplementary information, Table S7.

### Disease sensitivity and specificity of plasma cfDNA-derived 5hmC markers

We next assessed the ability of the 5hmC biomarkers derived from plasma cfDNA to classify cancer stages in a subset of patients with available records. The 5hmC classifier assigned incremental numbers of cancer individuals (predicted cancer probability > 0.5) for patients having undergone surgery for treatment (0/2), patients at cancer stage I (4/6) and patients at stage II and III (38/40) (Cochran-Armitage test for trend *P* = 0.00038, Figure 3F). The classifier had good but reduced power to call Stage IV patients (12/18), who in general suffered from metastasis to various tissues and are expected to have more complex tumor DNA profiles in circulation due to metastasis.

We further assessed disease specificity of the classifier in patients with colon-related benign diseases (*n* = 49) and patients with colorectal (*n* = 71), gastric (*n* = 61), liver (*n* = 25), pancreatic (*n* = 34) and thyroid (*n* = 46) cancer. Compared to the 86% call rate in colorectal cancer patients, only 8% of patients with benign colon diseases were predicted as having cancer (Figure 3G). The classifier also demonstrated certain tissue specificity, with a decreasing cancer call rate in gastric (85%), liver (44%), pancreatic (29%) and thyroid (28%) cancer patients (Figure 3G). The lower sensitivity in calling the other cancers is not due to any intrinsic difficulty in classifying those cancers, as we achieved a much greater sensitivity in liver and pancreatic cancer using 5hmC markers derived from plasma cfDNA from patients with these respective cancers (data not shown). These results indicate that distantly related cancers can be readily distinguished by the corresponding classifiers through joint testing, while classification of closely related cancers such as colorectal and gastric cancer may be facilitated by additional diagnostic criteria.

A subset of cancer patients had records of classical biomarkers and epidemiological risk factors (Supplementary information, Table S1), with which we compared plasma cfDNA-derived 5hmC biomarkers for cancer detection sensitivity. The detection sensitivity of carcinoembryonic antigen (CEA, 32%), alpha-fetoprotein (AFP, 0%), carbohydrate antigen 125 (CA125, 13%), CA15-3 (0%), CA19-9 (19%), CA72-4 (17%), cytokeratin 19 (49%), neuron-specific enolase (NSE, 21%), and relationship to overweight (body mass index ≥ 25 kg/m^2^, 34%), smoking (9%), alcohol consumption (7%) and previous history of cancer (0%) were all less than 50%. By calling cancer if any conventional biomarker or risk factor is positive, the upper bound detection sensitivity of the combined classical biomarkers and epidemiological factors only reached 54%, a sensitivity rate much lower than the 86% that we could achieve using 5hmC markers. In addition, compared with the methylated *SEPT9* (septin 9) gene, a blood-based epigenetic biomarker for colon cancer, our cfDNA 5hmC biomarkers registered a significantly further improved overall sensitivity (0.86 vs 0.48 based on public data)^32^.

### 5hmC markers derived from plasma cfDNA in gastric cancer

**Figure 4:**
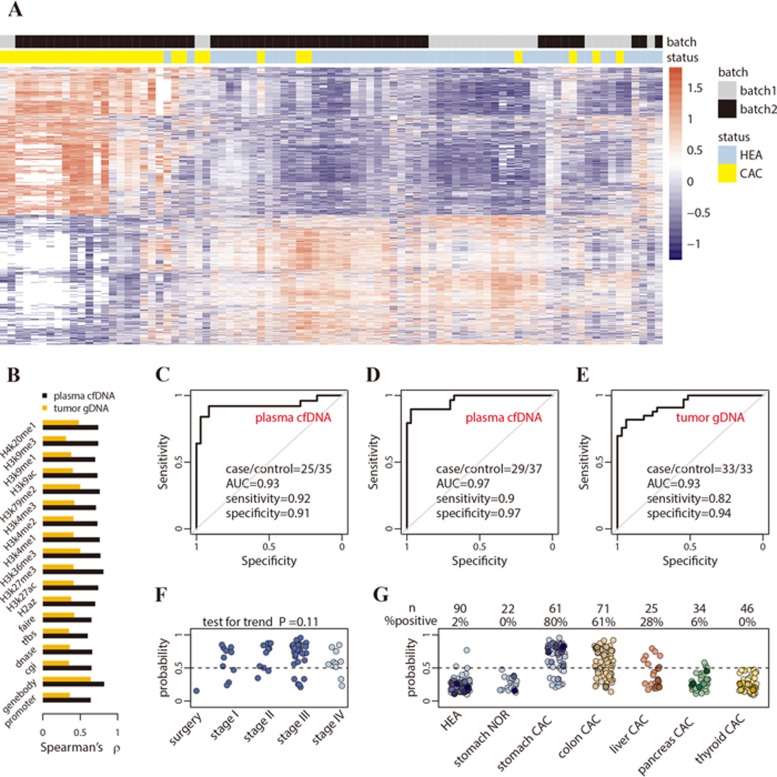
Performance of 5hmC biomarkers for gastric cancer. **(A)** The heat map shows clustering of cfDNA samples from both the discovery and validation batches, using the 1 431 differential gene bodies detected in plasma cfDNA from the discovery batch. **(B)** Correlation of 5hmC changes in cancer between the discovery and validation batches is higher in plasma cfDNA (cancer patients vs healthy individuals) than in tumor gDNA (tumors vs adjacent tissues), especially for 5hmC in gene bodies. **(C**, **D)** Classifying two independent validation batches using 5hmC classifier derived from plasma cfDNA from the discovery batch. **(E)** Classifying an independent set of gastric cancer tumor tissues using 5hmC biomarkers detected from the discovery batch of tissue samples (tumors vs adjacent tissues). **(F)** The predicted cancer probability (i.e., score) based on the 5hmC classifier from plasma cfDNA shows a trend associated with clinical stage. The one patient after surgery shows a predicted probability undistinguishable from healthy individuals. **(G)** The 5hmC cfDNA classifier for gastric cancer is disease- and potentially cancer type-specific, showing decreasing predicted probability in cfDNA from colorectal, liver, pancreatic and thyroid cancer patients.

Next, we analyzed gastric cancer using plasma cfDNA samples. In the discovery batch, 5hmC loci in 7 gastric cancer patients were compared to 18 healthy controls across genomic feature categories (Supplementary information, Table S4). Using the detected differential 5hmC loci, 25 gastric cancer patients could be generally separated from 35 healthy individuals in the validation batch (Figure 4A). Again, 5hmC changes in gene bodies showed relatively higher correlations between the discovery and validation batches compared to other genomic features (Figure 4B). A model-based classifier was generated using the 1 431 differential loci in gene bodies identified at 5% FDR and 1.2-fold change in the discovery batch (Supplementary information, Table S8). This was applied to the validation batch, achieving 92% sensitivity and 91% specificity (AUC = 0.93, Figure 4C). Further assessment of the gastric cancer classifier in an additional, independently collected validation batch (29 patients vs 37 controls) achieved 90% sensitivity and 97% specificity (AUC = 0.97, Figure 4D). The classification performance of the 5hmC biomarkers derived from cancer cfDNA was comparable to that from tumor gDNA samples: a classifier based on 161 differential 5hmC loci in gene bodies detected in 19 pairs of tumors and adjacent tissues in the discovery batch (Supplementary information, Table S9) was applied on 33 pairs of tissues in the validation batch, achieving 82% sensitivity and 94% specificity (AUC = 0.93, Figure 4E). The gastric cancer classifiers derived from plasma cfDNA and tissue gDNA profiles are detailed in Supplementary information, Table S10.

The 5hmC gastric cancer classifier derived from plasma cfDNA showed a trend of increasing cancer call rate with cancer clinical stage (*P* = 0.11, Figure 4F). The classifier also demonstrated disease and tissue specificity, with 0% cancer call rate for benign gastric diseases, and with decreasing cancer call rate in patients with colorectal (61%), liver (28%), pancreatic (6%) and thyroid (0%) cancer patients (Figure 4G).

The detection sensitivity of classical biomarkers and epidemiological factors for gastric cancer was 13% (CEA), 6% (AFP), 6% (CA125), 3% (CA15-3), 14% (CA19-9), 29% (CA72-4), 36% (cytokeratin 19), 13% (NSE), 18% (overweight), 25% (smoking), 10% (alcohol) and 7% (previous history of cancer) (Supplementary information, Table S1). The upper bound sensitivity combining these markers and factors was 70%, which again is lower than the 80% call rate observed in our studies.

### Tissue origin of the cancer-associated 5hmC changes observed in plasma cfDNA

**Figure 5:**
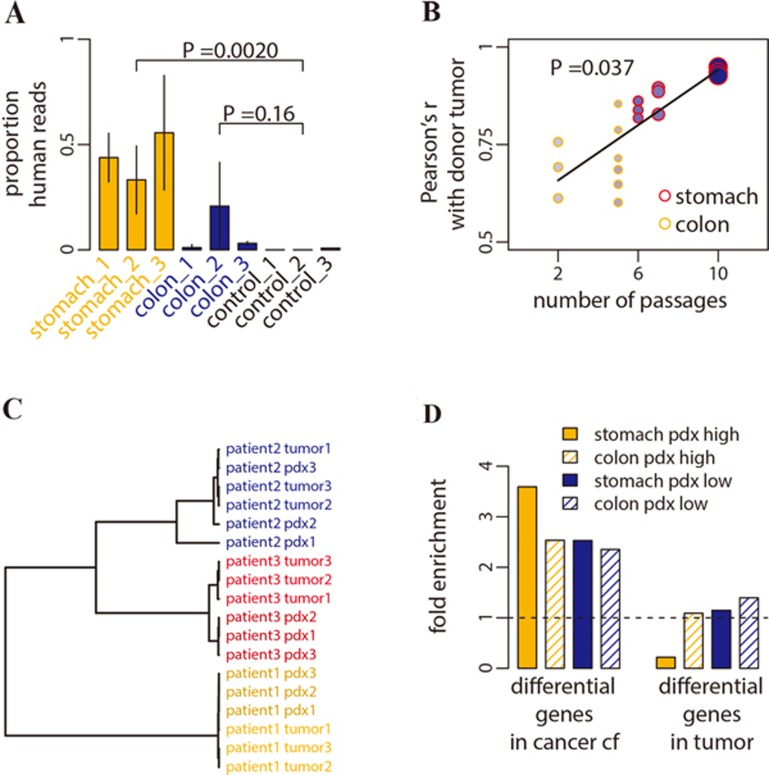
The origin of cancer-associated 5hmC changes observed in plasma cfDNA. **(A)** The proportions of human reads in plasma cfDNA captured with 5hmC-Seal are shown for PDX mice grafted with tumor from three gastric cancer patients (stomach_1-3), three colorectal cancer patients (colon_1-3) and for PDX mice without graft (control_1-3). Vertical bars represent s.d. estimated from three replicate PDX mice for each patient. The PDX mice grafted with gastric tumor had greater number of passages (6-10) than those grafted with colorectal tumor (2-5). **(B)** The correlation of the 5hmC profile between tumor-derived, PDX plasma cfDNA and donor tumor gDNA depends on the number of passages of the PDX mouse. The size of the points is proportional to the size of grafted tumor, and the density of color denotes the growth rate of the grafted tumor. **(C)** Using the correlation distance of the top five genes that had the greatest 5hmC level in PDX plasma cfDNA, donor tumor gDNA and PDX plasma cfDNA from the same individual patient were clustered together. **(D)** Genes with elevated 5hmC level in cancer patient plasma cfDNA are enriched in genes with high 5hmC levels in PDX plasma cfDNA. To estimate fold enrichment, the fifth percentile genes in descending order of the log_2_ fold change in patient cfDNA were compared against the corresponding fifth percentile genes in descending order of the 5hmC level in PDX mice (stomach pdx high, colon pdx high). Similarly, genes with decreased 5hmC level in cancer patient plasma cfDNA are enriched in genes with low 5hmC levels in PDX plasma cfDNA (stomach pdx low, colon pdx low). Dashed line denotes no enrichment.

To demonstrate the tumor relevance of the plasma cfDNA from cancer patients, we sought to examine its source in patient-derived xenograft (PDX) mouse models. PDX mouse models were derived from tumors of three colorectal patients and three gastric patients, each with three independent xenograft animals. Plasma cfDNA of PDX mice was collected at 12-15 weeks of age, from which the 5hmC-containing fragments were enriched and sequenced using the same protocol as with human plasma cfDNA. The proportion of cfDNA derived from the tumor, estimated as the proportion of sequencing reads uniquely mapped to the human genome, was significantly increased in mice grafted with gastric tumors (*P* = 0.0020) and showed an increased trend in mice grafted with colorectal tumors with fewer passages (*P* = 0.16, Figure 5A).

Only the sequencing reads mapped to the human genome were further analyzed. Pearson's correlation of 5hmC profile between plasma cfDNA of PDX mice and gDNA of donor tumors significantly depends on the number of passages (*P* = 0.037, Figure 5B). This suggests a quantitative relationship between tumor growth and the experimental capture of tumor 5hmC in plasma cfDNA, as the size (*P* = 0.0096) and growth rate (*P* = 0.0080) of tumors grafted in PDX mice increase with passage numbers (Figure 5B). Using the top five genes with the greatest 5hmC levels in PDX plasma cfDNA, donor tumor and the derived PDX from the same individual patient were clustered together (Figure 5C), indicating donor tumor tissue as the human origin of the PDX cfDNA.

PDX allowed us to study tumor-derived cfDNA without confounding background cfDNA. Genes with greater 5hmC levels in tumor-sourced PDX plasma cfDNA were more likely to be the genes with elevated 5hmC level in plasma cfDNA in cancer patients. Indeed, we found that genes with increased 5hmC levels in patient plasma cfDNA were enriched in those genes with greater 5hmC levels in PDX plasma cfDNA, whereas genes with decreased 5hmC levels in patient plasma cfDNA were enriched in genes with lower 5hmC levels in PDX plasma cfDNA (Fisher's exact tests *P* < 1×10^−9^, Figure 5D). In contrast, genes with 5hmC level changed between tumor and adjacent tissues showed no such enrichment pattern (Figure 5D).

### Tumor-associated 5hmC changes in gene regulation

To investigate the potential functional role of 5hmC in gene regulation, we evaluated the relationship between gene expression changes and 5hmC level changes in tumors in two colorectal and one gastric cancer patients. We performed an RNA-seq assay in tumor tissues and paired adjacent tissues. The log_2_ fold changes of gene expression and the log_2_ fold change of 5hmC level in tumors relative to adjacent tissues were estimated across the three patients. Gene dysregulation and 5hmC changes were then compared across a combined list of 200 differential 5hmC loci in gene bodies detected in colorectal and gastric tumors in the discovery batches. The correlation between gene expression changes and 5hmC changes in tumors is highly significant (*P* = 9.8 × 10^6^, Supplementary information, Figure S6A). In addition, genes with altered 5hmC levels in cancer plasma cfDNA or in tumor gDNA were enriched in cancer- and metastasis-related pathways^33^ (Supplementary information, Figure S6B).

## Discussion

In general, 5hmC marks active loci because gene activation requires removal of the repressive 5mC methylations. It occurs in gene bodies of activated genes as well as at various enhancers, indicating that the genomic locations of 5hmC reflect gene activation at permissive chromatin (Supplementary information, Figure S3). 5hmC is chemically stable and thus its locations in gDNA can be stored in fragmented cfDNA for potential detection using non-invasive sampling. Utilizing a robust and highly efficient profiling-based approach to map 5hmC in plasma cfDNA samples from patients with cancer, we were able to identify 5hmC biomarkers that can distinguish cancer patients from healthy individuals with high sensitivity and specificity for colorectal (Figure 3) and gastric (Figure 4) cancers. Our study revealed the genome-wide pattern of cancer-associated 5hmC changes in plasma cfDNA (Figure 2) and demonstrated its tumor origin using PDX models (Figure 5). The identified 5hmC biomarkers can be cancer type-specific (Figures 3 and 4). The optimization of this approach with additional patient studies in future will further improve its performance and expand the scope of its application to other cancers. The strategy presented here provides a foundation for effective future biopsy-based diagnosis using body fluids and potentially prognostic indicators of human disease progression using cfDNA.

The dynamics of cancer cfDNA turnover is yet largely unknown. Under what is likely to be a simplified model, gDNA of tumor tissue is released into plasma and undergoes degradation reaching an equilibrium similar to that of background cfDNA from normal healthy tissues. Locus-specific 5hmC modification appears to be the primary determinant of 5hmC levels, with tissue specificity and then the cancer state adding additional layers of variation. These tissue-, and to a lesser extent, cancer-specific signals in DNA released from tumor tissues slightly shift the 5hmC modification profile of background plasma cfDNA toward that of tumor tissue gDNA. The more cfDNA released from tumor tissues, the greater of the shift giving increased power to discriminate the biological and clinical variation of the tumor source. Therefore, it will be critical to integrate a panel of 5hmC profiles from gDNA of diverse tissue types to achieve future assessment of disease specificity with cancer biomarkers. In addition, solid tumors are composed of carcinoma stem cells and carcinoma cells, within a microenvironment constituted by leucocytes, cells of mesenchymal origin and extracellular matrix^34^. Tumor progression initiates a gradient of change in the local environment characterized by hypoxia and vascularization. Extensive variability may exist within a growing tumor and its surrounding cells such that certain types of cells are prone to apoptosis and to releasing DNA to the circulation. We expect that cancer-associated changes in 5hmC observed in plasma cfDNA were contributed by distinct sets of cells within or surrounding the tumor tissues. Single-cell or cell-type-specific 5hmC profiling of tumor-associated tissues and using appropriate cell type markers, would reveal the extent and distribution of the cell specificity of these modificaitons and shed further light on the properties of the source cells that contribute to the cancer-associated 5hmC changes observed in plasma cfDNA. It is our intent to pursue these future directions.

## Materials and Methods

### Study design

**Patient population** A total of 180 patients older than 18 years with colorectal (CC), gastric (GC) cancers and hepatocellular carcinoma were diagnosed at three different medical centers in Shanghai Huashan Hospital at Fudan University, China from September 2015 to July 2016. Eighty patients with thyroid cancer and pancreatic cancer were diagnosed at Peking Union Medical College Hospital, China during 2014-2016. The population was socioeconomically diverse, most patients coming from Beijing and East China (the City of Shanghai and the Provinces of Zhejiang, Jiangsu, and Anhui). All specimens were collected from patients who were newly diagnosed or having postoperative recurrence, as well as from two postoperative colorectal cancer patients and one postoperative gastric cancer patient, and had the tumor status confirmed by histological evaluation. Patients treated with chemotherapy, radiation therapy or immunotherapy were excluded from this study. In total, this study was conducted among 80 colorectal cancer patients and 75 gastric cancer patients, with an additional 25 hepatocellular carcinoma patients, 34 pancreatic cancer patients and 46 thyroid cancer patients to explore cancer type specificity. Whole blood samples from 90 healthy individuals under physical examination were also collected at Fudan University, China during September 2015-May 2016 as healthy controls; these individuals are Han Chinese and showed no history of cancer and had no abnormalities in laboratory examinations. However, the follow-up data for all patients were unavailable because of the short follow-up time. Informed consent was obtained from each participating subject before the study, which was approved by the Institutional Review Board (IRB) at each collaborating institution.

**Batch design** To minimize the influence of any batch effect, gastrointestinal participants were assigned into three batches in chronological order of enrollment in the study. Differential 5hmC features between cancer patients and controls were analyzed with batch 1 (discovery set) and validated in batches 2 and 3 (validation sets).

**Sample overview** Detailed information about the study subjects is shown in Supplementary information, Tables S1 and S2, including number of samples, gender, age, clinical diagnosis, stage classified according to the tumor, node and metastasis (TNM) guidelines (version 7), and eight conventional cancer biomarkers that were measured in patients. Four common tumor markers for gastrointestinal cancer screening are CEA, AFP, CA19-9 and CA72-4, which were positive in 0%, 33.8%, 21.4% and 16.9% of CC, as well as 7.5%, 13.6%, 14.3% and 29.0% of GC patients, respectively.

**Additional US samples for validation** Five patients with colorectal cancer were diagnosed at the University of Chicago Medical Center (UCMC) from September 2015 to July 2016. All samples were collected from patients who were newly diagnosed and had no distal metastasis at the time that blood was taken. Whole blood samples from six healthy individuals, who are non-Hispanic or Latino individuals of European ancestry, under physical examination were also collected at the UCMC during September 2015-May 2016 as healthy controls. Informed consent was obtained from each participating subject before the study, which was approved by the IRB at the University of Chicago.

### Preparation of cfDNA samples

cfDNA samples were prepared from peripheral blood collected from patients and healthy controls. Briefly, 4 ml of peripheral blood was collected from each subject using EDTA anticoagulant tubes, and the plasma sample was prepared within 6 h by centrifuging twice at 1 350× *g* for 12 min, and then centrifuging at 13 500× *g* for 12 min. The prepared plasma samples (about 2 ml/subject) were immediately stored at −80 °C. The plasma cfDNA was isolated using the QIAamp Circulating Nucleic Acid Kit (Qiagen) according to the manufacturer's protocol. Within each experimental batch, samples were randomized with respect to disease status in the following library preparation and sequencing profiling.

### Isolation of gDNA from tissues

Tissue samples, including tumor and adjacent tissue samples, from patients were stored at −80 °C after surgical removal. About 10-25 mg tissue was collected using a scalpel after sample thawing. gDNA from tissues was isolated using the ZR Genomic DNA Tissue Kits (Zymo Research) according to the manufacturer's protocol.

### 5hmC-Seal-seq library preparation and sequencing

Seal-seq libraries for 5hmC profiling were prepared following our previously patented technology^29^

In this method, the T4 bacteriophage β-glucosyltransferase is used to transfer an engineered glucose moiety containing an azide group onto the hydroxyl group of 5hmC across the human genome. The azide group is then chemically modified with biotin for affinity enrichment of 5hmC-containing DNA fragments. First, the gDNA is fragmented using an enzymatic reaction. Next, the fragmented gDNA or the cfDNA were repaired and installed with the Illumina compatible adaptors. The glucosylation reactions were performed in a 25 μl reaction containing 50 mM HEPES buffer (pH 8.0), 25 mM MgCl_2_, purified DNA, 100 μM N_3_-UDP-Glc and 1 μM βGT at 37 °C for 1 h. The reaction mix was subject to purification using a Micro Bio-Spin 30 Column (Bio-Rad) into ddH_2_O. Subsequently, 1 μl DBCO-PEG4-DBCO (Click Chemistry Tools, 4.5 mM stock in DMSO) was added to the reaction mixture. The reactions were incubated at 37 °C for 2 h. Next, the DNA was purified ona Micro Bio-Spin 30 Column (Bio-Rad). The purified DNA was incubated with 5 μl C1 Streptavidin beads (Life Technologies) in 2× buffer (1× buffer: 5 mM Tris pH 7.5, 0.5 mM EDTA, 1 M NaCl) for 15 min according to the manufacturer's instruction. The beads were subsequently washed eight times for 5 min with 1× buffer. All binding and washing was done at room temperature with gentle rotation. The captured DNA fragments were amplified with 14-16 cycles of PCR amplification. The PCR products were purified using AMPure XP beads according to the manufacturer's instructions. The DNA concentration of each library was measured with a Qubit fluorometer (Life Technologies) and sequencing was performed on the Illumina Hi-Seq or NextSeq 500 platform.

### RNA-seq library preparation and sequencing

Tumor and adjacent tissue samples including two colon samples and one stomach sample were collected and RNA was isolated using the ZR-Duet DNA/RNA Miniprep Kit (Zymo Research). Total isolated RNA was used to construct the library with the NEBNext Ultra RNA Library Prep Kit for Illumina following the manufacture's protocol. Sequencing reactions were executed on the NextSeq 500 platform using paired-end mode, yielding at least 32 M reads per sample.

### Establishment of patient-derived tumor xenografts

The animal protocol for this study was reviewed and approved by the Ethical Committee of Medical Research, Shanghai Huashan Hospital of Fudan University. It utilized 6-8 week old BALB/c nu/nu mice weighing 16-20 g on receipt (Shanghai Laboratory Animal Center). The fresh pathological tissue fragments were placed in sterile tissue culture medium on ice and brought immediately to the animal facility. Tumor graft samples were cut into multiple 1 × 1 × 1 mm fragments in complete media. Tumor material was implanted into female BALB/c nu/nu mice under isoflurane anesthesia; all possible efforts were made to minimize suffering. A skin incision (0.3 cm) was subsequently made on the right mid-back. One tumor piece (1-3 mm) was inserted into each pocket and the skin was closed. Mice were regularly checked. When thetumor diameter reached 1.5 cm, mice were euthanized and tumors were excised, cut into 1 × 1 × 1 mm fragments again, and passaged to successive generations of three mice. The remaining tumor was snap frozen in liquid nitrogen and stored at −80 °C; plasma was also separated from blood sampled via the mouse eyeball. In this study, the gastric cancer and colorectal cancer patient-derived tumor xenografts (PDX) were randomly selected in our existing PDX model library, while the control group was BALB/c nu/nu mice of 12-14 weeks old.

### 5hmC enrichment analysis

We designed two similar spike-in probes with unique sequences, named 5hmC spike-in and no5hmC spike-in.

5hmC spike-in:

5′-CTGTCATGGTGACAAAGGCATCC*GGCAGAAATGCCCACACAGCCTCTTTAACCAGCACGCC AACCGCCTCTGCTTCGGCCCTGGTCACGCAGCTGACAAGGTCTTCATAATAGAGAAATCCTG-3′, C* — 5hmC modifications.

no5hmC spike-in:

5′-CTGTCATGGTGACAAAGGCATCGCAGCGAAATGCCCACACAGCCTCTTTAACCAGCACGCC AACCGCCTCTGCTTCGGCCCTGGTCACGCAGCTGACAAGGTCTTCATAATAGAGAAATCCTG-3′

These sequences cannot map to the human reference genome. Six cfDNA sequencing libraries were constructed from the same cfDNA (10 ng) sample, which were divided into control and experimental groups, each having three duplicates. About 100 million copies of 5hmC and no5hmC spike-ins were then mixed with the experiment sample before library preparation. The control group did not include the 5hmC pull-down step, whereas the experimental group included the 5hmC pull-down procedure. After sequencing, we extracted spike-in reads and calculated the enrichment ratios. The average ratio of 5hmC spike-in to no5hmC spike-in in the control group was 0.72, while the ratio in the experimental group was 40.36.

### Technical stability analysis for 5hmC Seal-seq library preparation

The designed spike-in probes were utilized to improve the robustness and sensitivity of 5hmC-Seal. In total 20 000 copies of 5hmC and no5hmC spike-ins were pre-mixed and then added into the same cfDNA samples before constructing libraries. Different spike-in samples were designed as follows: 20 ng cfDNA with 2 repeats, 10 ng cfDNA with 10 repeats, 5 ng cfDNA with 2 repeats, 2 ng cfDNA with 2 repeats, 1 ng cfDNA with 2 repeats.

### Total 5hmC quantification in cfDNA and gDNA

The enzymatic digestion protocol for each gDNA and cfDNA sample was the same. gDNA or cfDNA (all in 8 μl H_2_O) was first denatured by heating at 95 °C for 5 min and then transferred into ice water for 2 min. Next, 1 L of 10× S1 nuclease buffer (30 nM CH_3_COONa, pH 4.6, 260 mM NaCl, 1 mM ZnSO_4_) and 180 units (1 μl) of S1 nuclease were added into the DNA solution. The mixture (10 μl) was then incubated at 37 °C for 4 h. Then 34.5 μl of H_2_O, 5 μl of 10× alkaline phosphatase buffer (50 mM Tris-HCl, 10 mM MgCl_2_, pH 9.0), 0.5 μl of alkaline phosphatase were added into the DNA digestion solution. The incubation was continued at 37 °C for an additional 4 h.

The CE-ESI-MS experiments were carried out with the CESI-8000 capillary electrophoresis (CE) system from Beckman Coulter coupled with a Sciex Tripel Quad 5500 Mass Spectrometer (Sciex) through a modified Nanosprayed II interface. Bare fused silica capillaries etched with porous tips were made available by Beckman Coulter; these could be inserted into the sheathless nanospray interface. The separation capillary was 100 cm long with an internal diameter of 30 μm and an outside diameter of 150 μm. The capillary was flushed with methanol for 10 min at 100 psi, followed by water, 0.1 M sodium hydroxide, 0.1 M hydrochloric acid and water for 10 min each at 100 psi, and finally by the background electrolyte (BGE) of 10 % acetic acid (pH 2.2) for 10 min at 100 psi before first use. The BGE was also used as conductive liquid in the conductive liquid capillary. Before each run, the conductive liquid capillary was rinsed with BGE for 5 min at 100 psi. Samples for detection were stored at 5 °C in the CE system. Hydrodynamic injections were used in this study, and about 100 nl sample was injected into the separation system for each analysis. A voltage of +25 kV was applied during the separation and the current was between 3.0 and 3.2 μA. The electrospray voltage was optimized to get the best nanospray stability and efficiency, and +1.7 kV was good enough for this study. The quantification calibration curves of 5′-dC, 5′-mdC and 5′-hmdC were constructed using mixture solution of their standards in different concentration. The resulting solutions of our DNA samples were directly measured by CE-ESI-MS. The concentrations of these three nucleosides in each sample were calculated based on the calibration curves. And the 5′-mdC/dC and 5′-hmdC/(dC + 5′-mdC) results of each sample were then calculated.

### Sequencing data processing and detection of differential loci

Read-through sequences within raw sequencing reads were trimmed using Trimmomatic version 0.35^35^. Low-quality bases at the 5′ (Phred quality score < 5) and 3′ (5 bp-sliding window Phred score < 15) were also trimmed. Reads with a minimum length of 50 bp were aligned to the human genome assembly GRCh37 using Bowtie2 version 2.2.6^36^ with the end-to-end alignment mode. For paired-end sequencing data, read pairs were concordantly aligned with fragment length < 500 bp and with up to 1 ambiguous base, and four mismatched bases per 100 bp length. Alignments with Mapping Quality Score (MAPQ) ≥ 10 were counted for overlap with genomic features using featureCounts of subread version 1.5.0-p1^37^, without strand information. Autosomal feature counts with > 10 mean counts across samples were then normalized and compared between-group using DESeq2 version 1.12.3^38^. Since gender is not a significant covariate for both autosomal gene expression^39^ and DNA methylation^40^, while aging has been linked to DNA methylation^41^, age at sample collection/surgery was included as a categorical variable (< 20, 20-55, > 55 yr) in the negative binomial generalized linear model implemented in DESeq2. As for the experimental batches (discovery and validation sets), samples were processed in less than 1 week by 1-3 technicians, the identity of the technician was included in the model to adjust for potential technical correlation. When comparing tumor and adjacent tissues, patient identity was nested under technician identity. A FDR^42^ of 5% was used to identify differential 5hmC loci.

For PDX mouse plasma cfDNA data, sequencing reads were trimmed and aligned to a composite assembly of mixed human and mouse (GRCm38) genome. Unique alignments (MAPQ ≥ 10) were separated into human and mouse reads by chromosome name.

For RNA-seq data, sequencing reads were trimmed and aligned to GRCh37 annotated with GENCODE release 19, using STAR version 2.5.1b^43^. Unique alignments with ≥ 90% match over reads were summarized by featureCounts. For the correlation analysis presented in Supplementary information, Figure S6A, 5hmC data were also summarized over exon regions as in RNA data. For genes having > 10 mean counts across samples, log_2_ fold change between tumor and adjacent tissues were estimated by DESeq2 adjusting for patient identity.

### Refining 5hmC biomarkers and evaluating performance

Cancer prediction models were trained using the differential 5hmC loci (e.g., gene bodies) detected in the discovery batch. We applied elastic net regularization on a logistic linear regression model^44^, using the *glmnet* library in the R Statistical Package^44^:


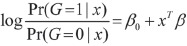


Where *x* is a *J* ∈ (1 ... *j*) by *I* ∈ (1 ... *i*) matrix of 5hmC level at gene *j* for sample *i*. The model is solved by


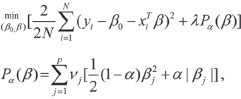


where *P_α_* is the blend of the ridge (*α* = 0) and the lasso (*α* = 1) penalty. The parameter *λ* controls for the overall strength of penalty, while the parameter *α* controls for the relative proportion between the ridge and lasso penalty. For a given *α*, *λ* is estimated by cross validation and selected as the largest *λ* at which the mean cross-validated error is within one s.e. of the minimum. As we expect that 5hmC loci with a larger effect size are more robustly detected by the assay and therefore more reproducible across experiments, we included in the model a penalty factor *ν_j_* so that


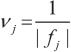


where *f_j_* is the log_2_ fold change of gene *j* estimated in the training batch.

The parameter *α* reflects the model assumption (i.e., a large number of small effects or a small number of large effects). In small data sets like our discovery batch, the selection of *α* based on residual errors may lead to an over-fitted model. Instead, our model assumption was guided by the validation batch, so that *α* was searched to maximize AUC in the validation batch over a grid of values from 0.05 to 0.95. The model, derived from the training batch using the selected α, was applied in all classifications as described in Figures 3 and 4.

The normalization of data in cancer classification adopts the regularized log transformation implemented in DESeq2, which estimates a global mean dispersion trend to shrink the variance at low count genes that are associated with high Poisson noise, so that variance is stabilized across genes in the log transformed data. In Figure 3 and Supplementary information, Figure S6, data from the validation batches were all normalized to the reference distribution derived from the training batch and used directly in cancer classification, i.e., we essentially ignored any remaining batch effect, which could result from library preparation and the sequencing run. This is because under a real clinical setting, the batch effect estimated between testing samples and training samples will generally be biased due to the highly unbalanced case/control proportion in testing samples (low incidence of cancers). Batch effect may introduce some deviation from the 0.5 probability cutoff in cancer calling. External spike-ins may be used to estimate batch effect in future investigations.

ROC curves^45^ were generated to evaluate the performance of a prediction algorithm, using the *pROC*^46^ library in the R package. Sensitivity and specificity were estimated at the score cutoff that maximizes the sum of sensitivity and specificity using the *ROCR*^45^ library in the R package.

### Statistical analyses

For Figure 5A, the *P*-value was estimated by a linear mixed effects model: proportion of human reads (square root transformed) ∼ xenograft status (none|colorectal|gastric) + γ (tumor donor identity) + ε, random effect γ was introduced to control for correlation among replicate xenografts. For Figure 5B, *P*-value was estimated by a linear mixed effects model: Pearson's *r* with tumor donor ∼ number of passages + γ (tumor donor identity) + ε.

### Annotating 5hmC loci with genomic features and functional analysis

The genomic features analyzed included promoters (3 kb upstream of gene start), gene bodies (gene start to stop sites annotated by GENCODE release 24)^47^, CpG islands (annotated CpG islands by UCSC Table Browser plus +/ 1 kb region), and the ENCODE^31^ features (DNase I hypersensitive sites, TF binding sites and histone modifications)^31^. Each type of ENCODE feature from the profiled ENCODE cell lines was integrated into a single list of features by collapsing overlapped and nearby (< 150 bp) peaks. The ENCODE features analyzed in the 5hmC genomic distribution (Supplementary information, Figures S2 and S3) were as originally annotated without collapsing, with 20 000 features randomly sampled in the interquartile size distribution for each feature category. Pathway enrichment analysis of genes with cancer patient-associated 5hmC loci were explored based on the Kyoto Encyclopedia of Genes and Genomes (KEGG)^48^ using the NIH/DAVID tool^49^.

### Data availability

All of the raw and processed data used in this study have been uploaded to the NCBI Sequence Read Archive (SRP080977) and Gene Expression Omnibus (GSE89570) depositories. The R code related to classifier detection and modeling is available upon request.

## Author Contributions

Conceptualization: YZ, WZ, CH and JL; Methodology: XL, YS, JN, FY, LW, GJ; Investigation: XL, YS; Formal analysis: XZ and WZ; Resources: ZL, JZ, WZ, DX, YW, YD, SY, JH, JS, HH, FL, LH, PW, XQ, MC, TZ, QL, MD, ZL, GC, KM, SA, MB; Writing original draft: WL, XZ, XL, LY, WZ and CH; Writing, review and editing, YZ and JL; Funding acquisition: YZ, WZ and CH.

## Competing Financial Interests

The University of Chicago has filed for patent protection on the original 5hmC-Seal technology in 2012. CH was one of the inventors. XL and YS are the shareholders of a company that has licensed the technology for clinical applications. The remaining authors declare that they have no competing interests.

## Supplementary Material

Supplementary information, Table S1 Clinical characteristics of colorectal and gastric cancer patients and healthy controls.

Supplementary information, Table S2 General characteristics of hepatocellular carcinoma, pancreatic cancer, thyroid cancer, gastric benign diseases, colorectal benign diseases, US colorectal cancer patients, and US healthy controls.

Supplementary information, Table S3 Summary of samples and the sequencing quality in 5hmC profiling.

Supplementary information, Table S4 Summary of differential 5hmC loci in colorectal and gastric cancer detected for each feature type.

Supplementary information, Table S5 Differential 5hmC loci in gene bodies detected at 5% FDR and 1.2 fold-change in the plasma cfDNA from discovery batch of colorectal cancer patients.

Supplementary information, Table S6 Differential 5hmC loci in gene bodies detected at 5% FDR and 1.2 fold-change in the tumor gDNA from discovery batch of colorectal cancer patients.

Supplementary information, Table S7 Colorectal cancer classifiers derived from plasma cfDNA and tissue gDNA profiles.

Supplementary information, Table S8 Differential 5hmC loci in gene bodies detected at 5% FDR and 1.2 fold-change in the plasma cfDNA from discovery batch of gastricl cancer patients.

Supplementary information, Table S9 Differential 5hmC loci in gene bodies detected at 5% FDR and 1.2 fold-change in the tumor gDNA from discovery batch of gastric cancer patients.

Supplementary information, Table S10 Gastric cancer classifiers derived from plasma cfDNA and tissue gDNA profiles.

Supplementary information, Figure S1 Technical validation of the modified hmC-Seal assay using spike-in probes containing 5hmC.

Supplementary information, Figure S2 Global 5hmC levels in plasma cfDNA and tissue gDNA.

Supplementary information, Figure S3 Genomic distribution of 5hmC detected in plasma cfDNA and tissue gDNA.

Supplementary information, Figure S4 The median distribution of 5hmC is similar between cancer and control.

Supplementary information, Figure S5 Counts per million reads at *SULF1* gene (plus +/−20kb region) in tissue gDNA of 11 colorectal cancer patients (subset of Figure 2B).

Supplementary information, Figure S6 Tumor associated 5hmC changes in gene regulation.
