# Supplementary material for: 5-Hydroxymethylcytosine signatures in circulating cell-free DNA as diagnostic biomarkers for human cancers
Source: Cell Res. 2017 Sep 19;27(10):1243–57. doi: 10.1038/cr.2017.121 (PMC5630683; doi:10.1038/cr.2017.121)
Supplement: Supplementary information, Table S1 — Clinical characteristics of colorectal and gastric cancer patients and healthy controls. [file cr2017121x1.pdf]

Table S1. Clinical characteristics of colorectal and gastric cancer patients and healthy controls.

	No. (%) or mean ± SD Participants of each group			P-value [a]	
	CC (n = 80)	GC (n=75)	HC (n =90)	CC vs. HC	GC vs. HC
**Age (years)**	60.78± 11.61	59.71±12.45	45±13.252	<0.001	<0.001
**Gender**					
**Male**	47(58.8)	47(62.7)	57(63.3)	0.7	0.9
**Female**	33(41.3)	28(37.3)	33(36.7)		
**BMI (kg/m^2)**	23.60±3.32	22.36±3.31			
**Smoking status**					
**Never**	64(90.1)	58(78.4)			
**Ever**	7(9.9)	16(21.6)			
**Drinking status**					
**Never**	66(93.0)	68(91.9)			
**Ever**	5(7.0)	6(8.1)			
**Tumor size,**					
**Greatest dimension (mm)**	39.99±15.75	44.2±26.889			
**Diagnosis**					
**Primary**	77(96.3)	73(97.3)			
**Postoperative recurrence**	1(1.2)	2(2.7)			
**Resection of tumors**	2(2.5)	0			
**TNM stages**					
**I**	5(6.5)	11(15.5)			
**II**	7(9.1)	10(14.1)			
**III**	43(55.8)	37(52.1)			
**IV**	22(28.6)	13(18.3)			
**Pathological grade**					

**Good**	4(6.8)	5 (7.8)		
**Moderate**	51(74.6)	12(18.8)		
**Low**	11(18.6)	47(73.4)		
**Depth of invasion**				
**T1**	4(6.3)	12(18.8)		
**T2**	7(10.9)	0(0)		
**T3**	2(3.1)	11(17.2)		
**T4**	51(79.7)	41(64.0)		
**Lymphatic metastasis**				
**Positive**	35(53.8)	39(62.9)		
**Negative**	30(46.2)	23(37.1)		
**Distal metastasis**				
**Positive**	22(28.2)	11(15.4)		
**Negative**	56(71.8)	60(84.5)		
**AFP**				
**Positive**	0(0)	4(7.5)		
**Negative**	74(100)	49(92.5)		
**CEA**				
**Positive**	24(33.8)	9(13.6)		
**Negative**	47(66.2)	57(86.4)		
**CA19-9**				
**Positive**	15(21.4)	9(14.3)		
**Negative**	55(78.6)	54(85.7)		
**CA72-4**				
**Positive**	11(16.9)	18(29.0)		
**Negative**	54(83.1)	44(71.0)		
**NSE**				
**Positive**	8(19.5)	6(13.0)		

**Negative**	33(80.5)	40(87.0)		
**CK19**				
**Positive**	17(42.5)	15(35.7)		
**Negative**	23(57.5)	27(64.3)		
**CA15-3**				
**Positive**	0(0)	1(2.8)		
**Negative**	48(100)	35(97.2)		
**CA125**				
**Positive**	6(11.3)	2(5.1)		
**Negative**	47(88.7)	37(94.9)		
**Collected sample No.**				
**Only Peripheral blood**	36(45.0)	23(30.7)	90(100)	
**Both peripheral blood and tissue**	37(46.25)	39(52.0)		
**Only tissue**	7(8.75)	13(17.3)		

[a] Chi-squared test.

Notes: CC, Colorectal Cancer; GC, Gastric Cancer; HC, Healthy Control; BMI: body mass index; NES: Neuron-Specific Enolase; CEA: carcinoembryonic antigen; AFP: alpha-fetoprotein; CA125: carbohydrate antigen 125; CA15-3: carbohydrate antigen 15-3; CA19-9: carbohydrate antigen 19-9; CA72-4: carbohydrate antigen 72-4.
