# Supplementary material for: 5-Hydroxymethylcytosine signatures in circulating cell-free DNA as diagnostic biomarkers for human cancers
Source: Cell Res. 2017 Sep 19;27(10):1243–57. doi: 10.1038/cr.2017.121 (PMC5630683; doi:10.1038/cr.2017.121)
Supplement: Supplementary information, Table S2 — General characteristics of hepatocellular carcinoma, pancreatic cancer, thyroid cancer, gastric benign diseases, colorectal benign diseases, US colorectal cancer patients, and US healthy controls. [file cr2017121x2.pdf]

Table S2. General characteristics of hepatocellular carcinoma, pancreatic cancer, thyroid cancer, gastric benign diseases, colorectal benign diseases, US colorectal cancer patients, and US healthy controls.

Cancer					Benign Diseases			US samples		
	No. (%) or mean ± SD Participants of Hepatocellular Carcinoma (n =25)	No. (%) or mean ± SD Participants of Pancreatic cancer (n =34)		No. (%) or mean ± SD Participants of Thyroid cancer (n=46)		No. (%) or mean ± SD Participants of Gastric Benign diseases (n =22)	No. (%) or mean ± SD Participants of Colorectal Benign diseases (n =49)		No. (%) or mean ± SD Participants of US colorectal cancer patients CC (n = 5)	No. (%) or mean ± SD Participants of US unaffected controls (n = 6)
**Age (years)**	59.32±11.86	53.06±13.04	**Age (years)**	41.75± 9.44	**Age (years)**	62.68±15.03	60.78±12.02	**Age (years)**	61.38± 10.72	33.16± 15.51
**Gender**			**Gender**		**Gender**			**Gender**		
**Male**	19(76.0)	17(50)	**Male**	8(25)	**Male**	14(63.6)	27(55.1)	**Male**	3(60.0)	3(50.0)
**Female**	6(24.0)	17(50)	**Female**	24(75)	**Female**	8(36.4)	22(44.9)	**Female**	2(40.0)	3(50.0)
**Smoking status**			**TSH**		**Smoking status**			**Race, Ethnicity**	5(100)	6(100)
**Never**	12(52.17)	8(72.73)	**Lower than the normal value**	3(12)	**Never**	20(90.9)	45(91.8)	**White, Not Hispanic or Latino**		
**Ever**	11(48.73)	3(27.27)	**Higher than the normal value**	2(8)	**Ever**	2(9.1)	4(8.2)			
			**The normal value**	20(80)						
**Drinking status**			**FFT3**		**Drinking status**			**Smoking status**		
**Never**	14(60.87)	9(81.82)	**Lower than the normal value**	0	**Never**	22(100)	47(95.9)	**Never**	1(20.0)	4(66.7)
**Ever**	9(39.13)	2(18.18)	**Higher than the normal value**	0	**Ever**	0	2(4.1)	**Ever**	4(80.0)	2(33.3)
			**The normal value**	25(100)						
**CEA**			**FFT4**		**BMI (kg/m^2)**			**TNM stages**		
**Positive**	4(16.7)	1(11.11)	**Lower than the normal value**	0		22.90±3.16	22.71±3.11	**I**	0(0)	
**Negative**	20(83.3)	8(88.89)	**Higher than the normal value**	0				**II**	2(40)	
			**The normal value**	25(100)				**III**	2(40)	
								**IV**	1(20)	

**CA19-9**			**Lymphatic metastasis**		**Diagnosis**			**Depth of invasion**		
**Positive**	12(50)	3(37.5)	**Positive**	16		4(18.2)	41(83.7)	**T3**	3(60.0)	
**Negative**	12(50)	5(62.5)	**Negative**	2	**Gastritis or enteritis**	12(54.5)	8(16.3)	**T4**	2(40.0)	
					**Polyps**	6(27.3)	0			
					**Ulcers**					
**AFP**								**Lymphatic metastasis**		
**Positive**	14(58.33)	0						**Positive**	2(40.0)	
**Negative**	10(47.67)	5(100)						**Negative**	3(60.0)	
**Tumor size,**								**Distal metastasis**		
**Greatest dimension (mm)**	42.83±39.634							**Positive**	1(20.0)	
								**Negative**	4(80.0)	
**Number of tumor foci**										
**Singe**	13(56.52)									
**Multiple**	10(43.48)									
**HBsAg**										
**Positive**	20(83.33)									
**Negative**	4(16.67)									
**HBsAg (+) HBV-DNA**										
**≥ 500 IU/mL**	12(70.59)									
**< 500IU/mL**	5(29.41)									

Notes: CEA: carcinoembryonic antigen; AFP: alpha-fetoprotein; CA19-9: carbohydrate antigen 19-9.
