# Supplementary material for: 5-Hydroxymethylcytosine signatures in circulating cell-free DNA as diagnostic biomarkers for human cancers
Source: Cell Res. 2017 Sep 19;27(10):1243–57. doi: 10.1038/cr.2017.121 (PMC5630683; doi:10.1038/cr.2017.121)
Supplement: Supplementary information, Figure S1 — Technical validation of the modified hmC-Seal assay using spike-in probes containing 5hmC. [file cr2017121x11.pdf]

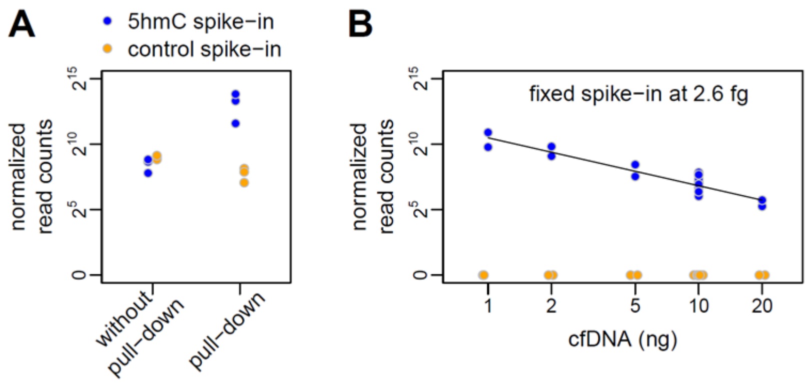

Figure S1 Technical validation of the modified hmC-Seal assay using spike-in probes containing 5hmC. **(A)** Enrichment of 5hmC by the pull-down assay. **(B)** Different amounts of cfDNA with fixed spike-in probes. The $\log_2$ cfDNA concentration and the mean $\log_2$ spike-in copy number at each concentration was close to a complete correlation (r^2=0.99). Note that technical replicates, including 10 spike-in replicates with 2.6 fg spike-in probes and 10 ng cfDNA performed by different individuals using different reagent batches, constituted 12% of total variance, further validated the robustness of this 5hmC-based approach using plasma cfDNA. In (A) and (B), cfDNAs of assay samples were derived from the same biological sample. Equal copies of two spike-in probes (5hmC-containing spike-in and control non-5hmC spike-in) were added to each assay sample. cfDNA together with spike-in probes were sequenced on the NextSeq 500 platform using paired-end 150 bp mode. The number of reads mapped to the sequence of the 5hmC-containing spike-in probe (blue) and control non-5hmC spike-in probe (orange) were counted.
