# Supplementary material for: 5-Hydroxymethylcytosine signatures in circulating cell-free DNA as diagnostic biomarkers for human cancers
Source: Cell Res. 2017 Sep 19;27(10):1243–57. doi: 10.1038/cr.2017.121 (PMC5630683; doi:10.1038/cr.2017.121)
Supplement: Supplementary information, Figure S2 — Global 5hmC levels in plasma cfDNA and tissue gDNA. [file cr2017121x12.pdf]

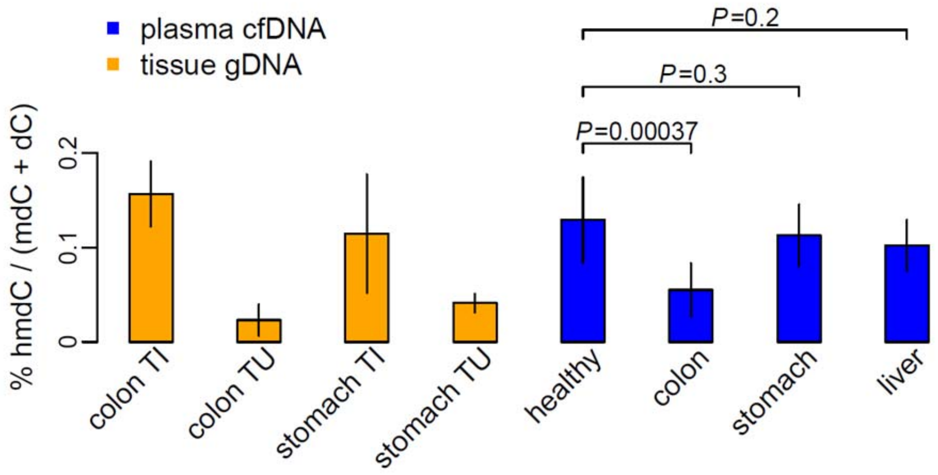

Figure S2 Global 5hmC levels in plasma cfDNA and tissue gDNA. P values were estimated for percent hmdC / (mdC+dC) in cancer cfDNA vs. control cfDNA, by a linear model: % hmdC / (mdC+dC) ~ cancer type (none | colorectal | gastric | liver) + age + gender + cfDNA concentration + experimental batch + ε. TI: tumor adjacent tissue; TU: tumor.
