# Supplementary material for: 5-Hydroxymethylcytosine signatures in circulating cell-free DNA as diagnostic biomarkers for human cancers
Source: Cell Res. 2017 Sep 19;27(10):1243–57. doi: 10.1038/cr.2017.121 (PMC5630683; doi:10.1038/cr.2017.121)
Supplement: Supplementary information, Figure S3 — Genomic distribution of 5hmC detected in plasma cfDNA and tissue gDNA. [file cr2017121x13.pdf]

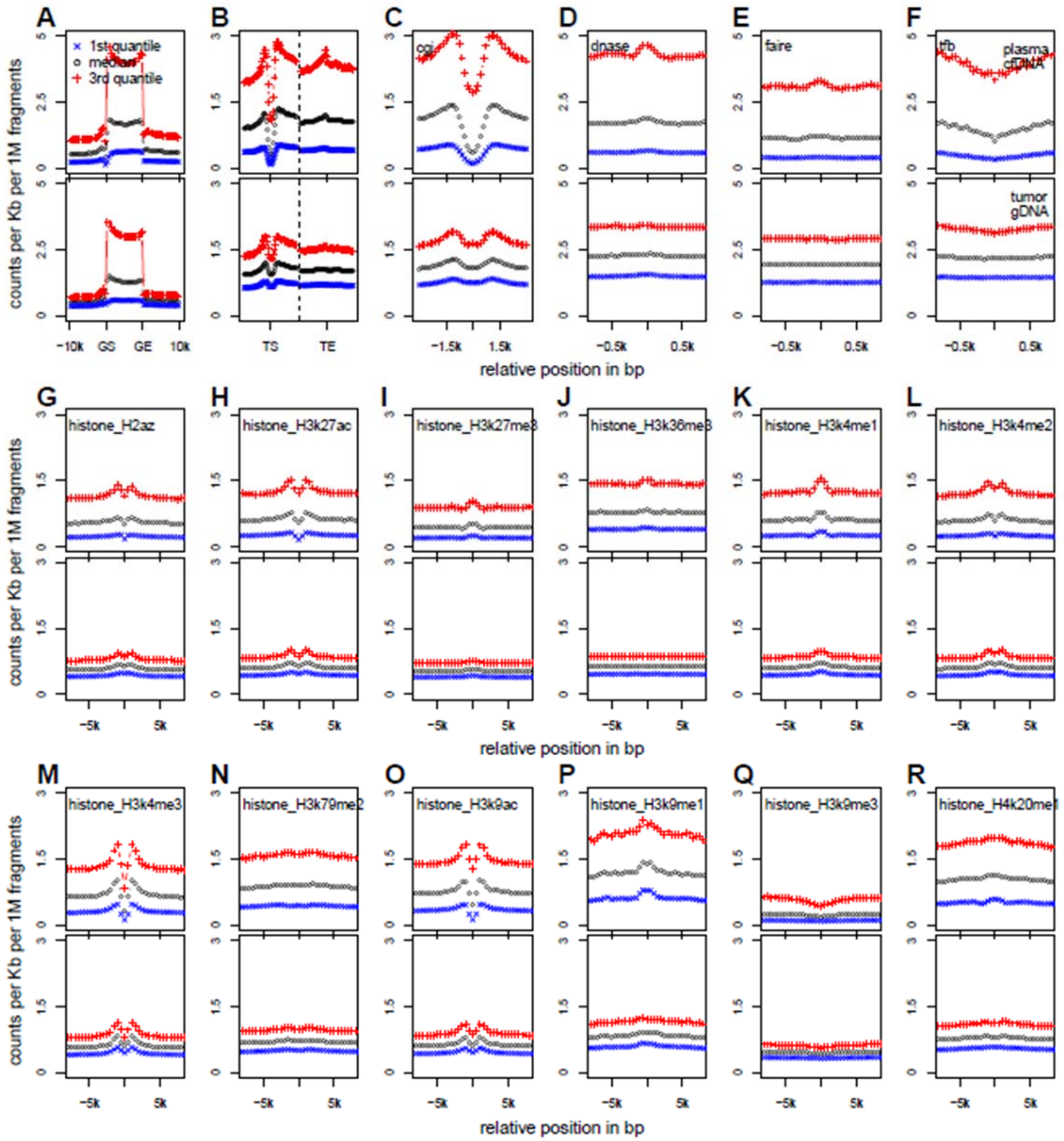

Figure S3 Genomic distribution of 5hmC detected in plasma cfDNA and tissue gDNA. The pooled results from the discovery batch of colorectal and gastric cancer patients as well as healthy controls are shown. (**A**-**R**) shows the distribution of each feature category, respectively, for plasma cfDNA samples (upper panels) and tissue gDNA samples (lower panels). **(A)** Gene bodies, defined by gene start (GS) and end (GE) sites, were divided into 20 positional bins; **(B)** Regions flanking transcription start (TS) or end (TE) sites; **(C)** CpG islands; **(D)** DNase I hypersensitivity peaks; **(E)** Formaldehyde-assisted isolation of regulatory elements; **(F)** Transcription factor binding peaks; **(G)** H2A.Z variant; **(H)** H3K27ac; **(I)** H3K27me3; **(J)** H3K36me3; **(K)** H3K4me1; **(L)** H3K4me2; **(M)** H3K4me3; **(N)** H3K79me2; **(O)** H3K9ac; **(P)** H3K9me1; **(Q)** H3K9me3; and **(R)** H4K20me1. In (C-R), equal-sized bins were centered at the feature center and extended to up- and down-stream.
