# Supplementary material for: 5-Hydroxymethylcytosine signatures in circulating cell-free DNA as diagnostic biomarkers for human cancers
Source: Cell Res. 2017 Sep 19;27(10):1243–57. doi: 10.1038/cr.2017.121 (PMC5630683; doi:10.1038/cr.2017.121)
Supplement: Supplementary information, Figure S4 — The median distribution of 5hmC is similar between cancer and control. [file cr2017121x14.pdf]

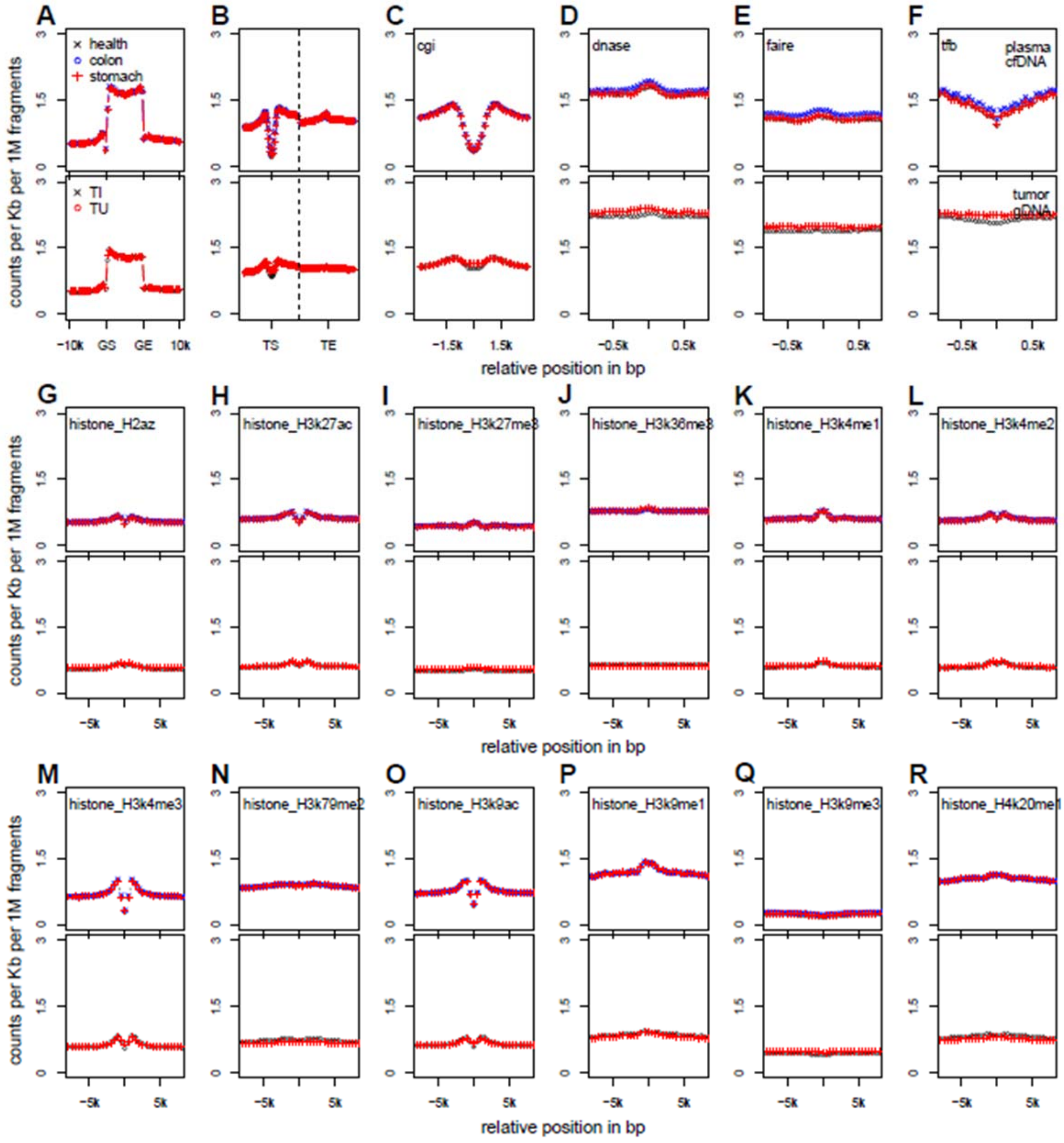

Figure S4 The median distribution of 5hmC is similar between cancer and control. (**A-Q**) shows the distribution of each feature category, respectively, for plasma cfDNA samples (upper panels) and tissue gDNA samples (lower panels). **(A)** Gene bodies, defined by gene start (GS) and end (GE) sites, were divided into 20 positional bins; **(B)** Regions flanking transcription start (TS) or end (TE) sites; **(C)** CpG islands; **(D)** DNase I hypersensitivity regions; **(E)** Formaldehyde-assisted isolation of regulatory elements; **(F)** Transcription factor binding peaks; **(G)** H2A.Z variant; **(H)** H3K27ac; **(I)** H3K27me3; **(J)** H3K36me3; **(K)** H3K4me1; **(L)** H3K4me2; **(M)** H3K4me3; **(N)** H3K79me2; **(O)** H3K9ac; **(P)** H3K9me1; **(Q)** H3K9me3; and **(R)** H4K20me1. In (C-R), equal-sized bins were centered at the feature center and extended to up- and down-stream.
