# Supplementary material for: 5-Hydroxymethylcytosine signatures in circulating cell-free DNA as diagnostic biomarkers for human cancers
Source: Cell Res. 2017 Sep 19;27(10):1243–57. doi: 10.1038/cr.2017.121 (PMC5630683; doi:10.1038/cr.2017.121)
Supplement: Supplementary information, Figure S5 — Counts per million reads at SULF1 gene (plus +/−20kb region) in tissue gDNA of 11 colorectal cancer patients (subset of Figure 2B). [file cr2017121x15.pdf]

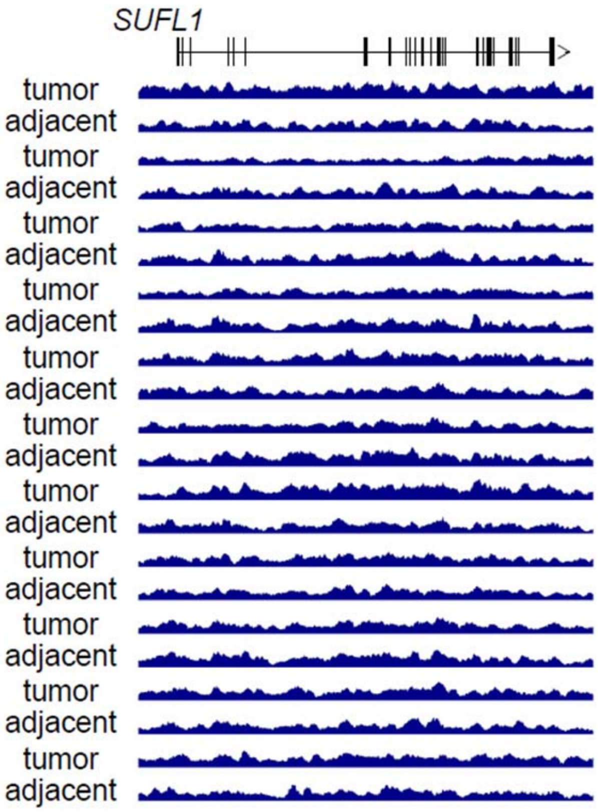

Figure S5 Counts per million reads at *SULF1* gene (plus +/-20kb region) in tissue gDNA of 11 colorectal cancer patients (subset of Figure 2B). There is no significant 5hmC level difference at *SULF1* between tumor and adjacent tissues. The moving averages at 0.01 smoother span are shown.
