# Supplementary material for: 5-Hydroxymethylcytosine signatures in circulating cell-free DNA as diagnostic biomarkers for human cancers
Source: Cell Res. 2017 Sep 19;27(10):1243–57. doi: 10.1038/cr.2017.121 (PMC5630683; doi:10.1038/cr.2017.121)
Supplement: Supplementary information, Figure S6 — Tumor associated 5hmC changes in gene regulation. [file cr2017121x16.pdf]

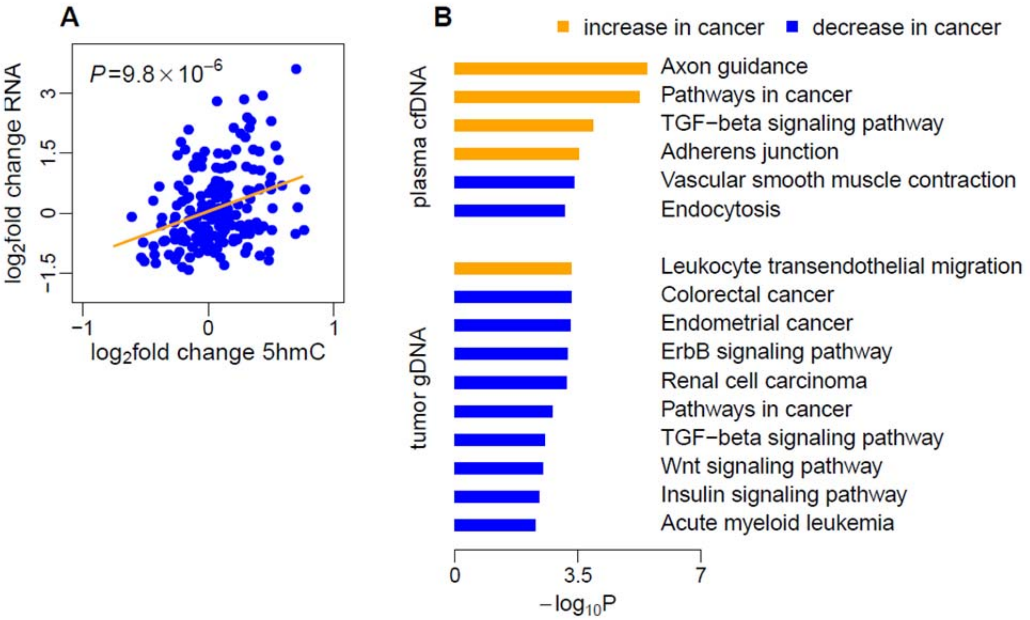

Figure S6 Tumor associated 5hmC changes in gene regulation. **(A)** Three pairs of tumors (2 colorectal and 1 gastric) and adjacent tissues were profiled by RNA-Seq. The $\log_2$ fold changes of gene expression in tumor vs. adjacent tissues were compared to the $\log_2$ fold change of 5hmC in tumor vs. adjacent tissues estimated for these three patients. Gene expression changes in the tumor is positively associated with 5hmC changes in the tumor for 200 differential 5hmC loci at gene bodies detected in either colon or stomach tumors in the discovery batches. **(B)** Gene pathway analysis of colorectal cancer-related canonical pathways using the NIH/DAVID tool.
